# An Empirical Study on Imagery and Emotional Response in Chinese Poetry Translation—The Visual Grammar Perspective

**DOI:** 10.3389/fpsyg.2022.872497

**Published:** 2022-07-12

**Authors:** Yuan Yuan, Tu Guoyuan

**Affiliations:** ^1^School of Foreign Languages, Central South University, Changsha, China; ^2^Faculty of Foreign Languages, Ningbo University, Ningbo, China

**Keywords:** mental imagery, poetry translation, reader's cognition, visual grammar, visual image

## Abstract

The study investigated the evocation of mental imagery and emotional responses when English–Chinese bilinguals read classic Chinese poems and their English translations to examine (1) the target readers' formation of non-verbal text representations of Chinese poetry and (2) whether different translations affect the target readers' imagery cognition. A total of 20 English–Chinese speaker students enrolled in a Chinese university read a classic Chinese poem in Chinese and its four versions of translation in English. Through questionnaires and interviews, participants rated the visualized words used in the poems for the degree of mental imagery and emotional response evoked based on three indicators of narrative process, salience value, and emotive validity in the theoretical framework of visual grammar. Results showed considerable individual variances in the cognitive differences in forming mental imagery in all versions of the poems and there were also effects of translation strategy. Moreover, visual language information in poetry reading and its translations evoked different emotional responses depending on the use of visual words with cultural features. Our study demonstrates the applicability and accessibility of visual language in describing different readers' mental imagery and the interrelation and interaction between the poetry language system and the emotional, social, and cultural contexts involved in poetry translation.

## Introduction

Mental imagery is a cognitive process that involves forming mental representations and images (Richardson, 1969; Kosslyn, 1994; Guarnera et al., 2019). The quasi-sensory and quasi-perceptual experiences in mental imagery can be formed without external stimuli that physically produce those sensory and perceptual experiences (Nanay, 2010). As the core of traditional Chinese aesthetics, mental imagery is a literary device widely applied in classic poetry in which vivid words are used to evoke an image or concept in the reader's mind. Through these words, poets seek to elicit emotional responses, rather than just painting a picture in the reader's mind, so that the imagery becomes a source of pleasure to readers (De Koning and van der Schoot, 2013). Empirical studies have examined the psychological and aesthetic responses to mental imagery during literary reading in both monolingual and bilingual contexts (Gunston-Parks, 1985; Goetz et al., 1993; Sadoski et al., 2000; Tierney and Readence, 2000; Sadoski, 2001). For instance, studies have shown the effects of imageability levels in mental imagery on single word processing in the form of both processing speed and brain activity (Steffensen et al., 1999; Ehlers-Zavala, 2005; Krasny and Sadoski, 2008; Connell and Lynott, 2012). Some evidence has also revealed contributions of different narrative styles to forming mental imagery elicited by a natural string of words (Magyari et al., 2020). However, translating imagery words from one language to another involves more complex cognitive operations than reading imagery words in either the native language or the second language. The cognitive operations underlying translating highly imageable and emotional poetry remain a topic unexplored.

In literary works, imagery is often considered a static visual description, i.e., a rendition of the story world wherein objects are ascribed visual properties but are separated from the characters' interactions with them (Wolf, 2004). However, Halliday (1967) argued that language is a social sign, “an atent system of meaning” and “the grammar of language” is not a set of systems but a resource for making meaning. Based on Halliday (1978)'s study on the social semiotic perspective, Kress and Van Leeuwen (1996, 2021) incorporated and extended the meta-functional theory (i.e., ideational function, interpersonal function, and textual function) by applying it to the level of visual modality to create a visual grammar, which interprets how images express various types of meanings (e.g., symbolic meaning, interactive meaning, and meaning of the composition). As Kress and Van Leeuwen (2021) put it, “grammar of the visual describes how depicted elements—people, places, and things—combine in the visual statements of greater or lesser complexity and extension”.

In recent years, research in visual grammar has primarily developed in three perspectives: first, audience studies such as corpus studies or eye-movement experiments to confirm (or falsify) an argument (Holsanova, 2012; Bateman, 2014); second, theoretical development and innovation that integrate different disciplines, revise and supplement the visual grammar theory based on corpus analysis and propose new theoretical frameworks (e.g., Bateman, 2008; Painter et al., 2013); third, multi-modal discourse analysis through authentic images or pictures (Serafini, 2011; Feng and O'Halloran, 2012; Foncubierta-Rodríguez et al., 2017; Teng and Miao, 2018). Most multi-modal translation studies focus on non-verbal texts, but little attention has been paid to pure verbal literary texts. The current study attempts to employ visual grammar as a theoretical framework. It focuses on three critical indicators—narrative process (i.e., identification of narrative structures), salience value, and emotive validity—to examine readers' perceptions of imagery in poetry translation. Narrative structures refer to the structure which represents aspects of reality in terms of unfolding actions and events, processes of change, transitory spatial arrangements, and so on (Kress and Van Leeuwen, 2021). Narrative structures can generally be categorized into action process, reactional process, and speech and mental process. In the “action process,” the participant who sends the vector is the actor, and the participant to whom the vector is directed is the goal. When the vector is formed by an eyeliner, by the direction of the glance of one or more of the participants, the structure is reactional. A special kind of vector in “speech and mental process” is formed by the thought bubbles and dialogue balloons in the image that connect drawings of speakers or thinkers to their speech or thought. Salience is one of the principles used to represent the compositional meaning of the image (Kress and Van Leeuwen, 1996). It refers to the elements (participants as well as representational and interactive system) that are made to attract the viewer's attention to different degrees, as realized by such factors as placement in the foreground or background, relative size, contrasts in tonal value (or color), the difference in sharpness, and so on (Kress and Van Leeuwen, 2021). Poetry writing has more opportunities to convey salience through the use of visual words and rhythmic features. The term “validity” encompasses what seem to be different types of truth that are realized in different semiotic modes, and expresses the social semiotic core idea that modality is based on the values, beliefs, and social needs of social groups. Abstraction and amplification of validity are described by abstraction and amplification of validity markers (Kress and Van Leeuwen, 2021). These markers can be various and abundant visual words that enhance the emotive attractiveness of the poems.

We also investigate how bilingual readers respond to and accept imagery by comparing their emotional reactions when reading the original and translated poems. Imagery in poetry is a real art sublimated from everyday life that begins with sensory contact and is subsequently processed and formed by a poet (Qu, 2002). A sinologist, Waley (2000), argues that imagery is the soul of poetry. Pound (2004), an American poet, believes that imagery can convey intellectual and emotional depth quickly in a poem or literary work. In literature, imagism emphasizes the union of emotion and form. According to Langer (1957), literature is a symbolic manifestation of human feelings, and the artistic symbol is the ultimate imagery full of passion, life, and personality. Imagery serves as the vehicle by which the poet expresses his or her emotional state, and the poet's rich emotional experience pervades the reproduction of imagery in literary translation. Therefore, the imagery reproduced must also be the reproduction of emotion, for only imagery with nearly the same emotional effect can faithfully convey the poet's thoughts and feelings while also reflecting the aesthetic value of the original poem in order to achieve its second life (Rojo et al., 2014). The purpose of the current study is to comment on poetry translation beyond the verbal system and highlight the importance of imagery creation, aesthetic experience, and cultural connotation in poetry translation.

Although the imagery word in poetry is difficult for readers to comprehend, particularly those with various cultural backgrounds, it has attracted universal attention and concern from poets and researchers. Cognitive and emotional investigation of mental imagery can help better understand how the text and the non-verbal system (mental imagery) work together in poetry translation, as well as how the source poem and the target poem are linked and reformed at the mental imagery level.

**Figure 1:**
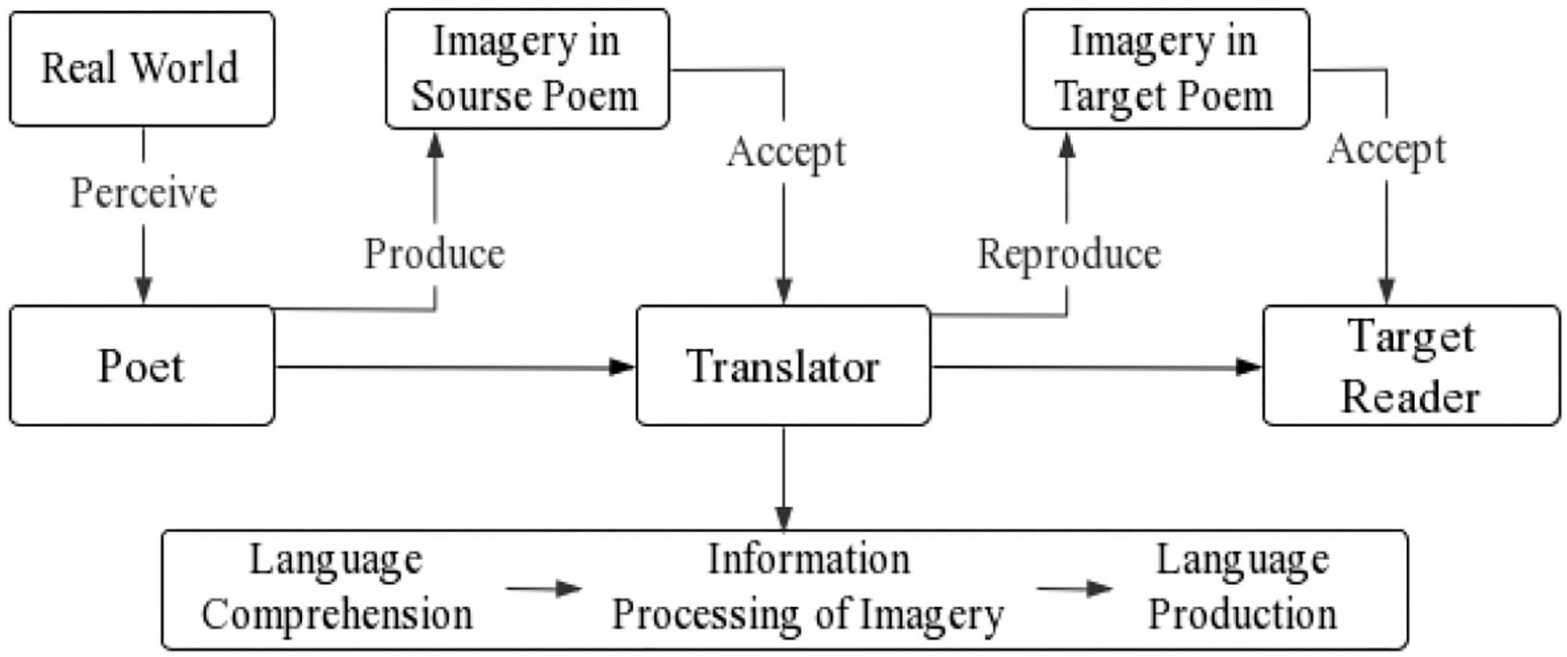
The cognitive process of poetry imagery translation.

The poet, the translator, and the target reader are all involved in poetry translation. The perception and interpretation of the imagery vary as the participants' identities alter during the cognitive process of poetry translation. The poet creates imagery in the poem based on real-world objects and the poet's own experiences and emotions. The translator accepts and transforms the imagery by reproducing it in the target language for the target reader (see Figure 1). The translator's cognitive process includes language comprehension, information processing of mental imagery, and language production, in which the translator has to determine the literal, emotional, and cultural meaning of the visual words in the source poem while perceiving the same aesthetic experience as the poet. Then the translator must search for equivalent words with the imagery effect in the target language for the target reader. The translator and the target reader might be thought of as recipients of the source and target poems. Therefore, the whole process of translating imagery contents in a poem involves the transition from visual symbols (words) to imagery, then from imagery to symbols (words in the target language), and finally from symbols to imagery on the part of the target reader.

## Methods

### Subjects

Twenty participants (7 females, 13 males), including senior and post-graduate international students currently studying at a Chinese university, participated in the study. Twelve participants speak English as their native language, and eight are fluent in English (L1). All of them speak Chinese as their second language (L2), and 15 of them have passed Level V of the HSK (Chinese Proficiency Test).^1^ All participants met or exceeded college curricular expectations for reading and writing in the Chinese language. Participation in the study was completely voluntary.

### Materials

A classic Chinese poem and its four versions of translations were used as materials. The title of the poem is “天净沙·秋思” in Chinese. The poem, which contains 28 Chinese characters, was written by Ma Zhiyuan in the Yuan Dynasty (1271–1368). It sketches a picture of a journey at dusk in an autumn field, enumerating the spatial imagery of nine scenes in the landscape of ancient Chinese villages. There are eight noun phrases with the “modifier-head” structure, divided into three groups, each composed of three visual words. On the surface, there appears to be no link between the nine scenes, but the context of the poem reveals that all of the scenes are utilized to portray a profound and emotional love for one's birthplace. The four versions of English translations are titled “Autumn”, “Tune: Tian Jin Sha”, “Tune to “Sand and Sky”-Autumn Thoughts”, and “Autumn Thoughts” by translators Weng Xianliang, a Chinese translator (Weng, 1978), Wayne Schlepp, a sinologist (Wen, 1989), Zhao Zhentao, a Chinese translator (Gu, 1993), and Ding Zuxin, a Chinese translator, and Burton Raffel, an American writer, translator (Ding and Raffel, 1992), respectively.

### Procedure

All participants were first familiarized with the concept of visual grammar and its three critical indicators, “narrative structure”, “salience value”, and “emotive validity” in the theoretical framework. Each participant was then given five copies of the poem, one original Chinese version and its four versions of English translations. Participants were asked to read the materials and filled a questionnaire regarding the observed visual images presented by the words used in the materials. An experimenter then conducted an interview with each participant to collect explanations and reasons for the participant's answers to the questionnaire and the cognitive process behind it. According to the previous empirical study on mental imagery in literary translation and visual grammar (Chen and Li, 2021), the questionnaire was divided into four parts. In the first part, participants needed to divide the poem (and its four versions of translations) into pauses (i.e., sections) by finding the visual words and forming imagery. In the second part, participants needed to describe the type of defined mental imagery using the narrative structure categorization. In the third part, the subjects needed to determine the salient elements they find at first sight and describe the ways to achieve the imagery's meaning based on the content of the salient values. Finally, participants had to report any mental imagery that evoked emotional reactions. At the same time, they read the poem and explain the connections between the imagery and emotion. During the survey questionnaire, participants could look up information about the visual words to ensure their choices.

## Results

### The Identification of Visual Images

After reading the original (source) poem (i.e., ST) and its four versions of English translations (i.e., TT1, TT2, TT3, and TT4), 15 participants identified 12 separate images in the source poem, TT2, and TT3. Sixteen reported 13 images in TT1, involving one visual word, “the far bank” which does not exist in the source poem. For TT4, sixteen participants identified the same 12 images as ST, but four participants identified more than 12 images. These four participants explained that “returning crows” and “at dusk” formed two separate images in their mental imagery and the same as “a narrow bridge” and “below the bridge.”

However, single visual pictures must be connected to disclose the poem's idea and produce an emotional and aesthetic experience on the reader's part. For example, the same linguistic construct (i.e., a modified and a noun) was used to communicate three images in the first three lines of the source verse, which might be connected to make more significant pictures. Participants were asked to combine those images in each line and report whether or not they could “see” the pictures or determine the representational meaning of imagery superimposition. As can be seen in Figure 1, the connection of the three images in Line 1 is the weakest among the three lines. Only six participants suggested that they could think of pictures about the season or the desert, which are the target meaning of the verse. The remaining 14 participants could not even find any connections between the images. Nevertheless, both Line 2 and 3 produced imaginative pictures, especially in TT1 and TT4. Much like linguistic structures, visual structures point to particular interpretations of experience and particular forms of social interaction (Kress and Van Leeuwen, 1996). Without cultural accumulation, the effect of the imagery superimposition of parallel nouns (such as withered vine, old tree, and crow at dusk in Line 1 of ST and TT3) could not be easily achieved. However, in target poems, it is possible to find how these images can be linked to each other. Some are linked in spatial, locative terms, such as Line 2, “Yonder is a tiny bridge over a sparkling stream, and on the far bank, a pretty little village” (TT1), and “A few houses hidden past a narrow bridge, and below the bridge a quiet creek running” (TT4). All participants suggested that these words evoked imagery of pastoral life in their minds. Verbs can relate to others, such as Line 3, “west wind moaning, horse groaning” (TT1), and “A lean horse comes plodding” (TT4), which formed imagery of a very burdened, tired horse.

**Figure 2:**
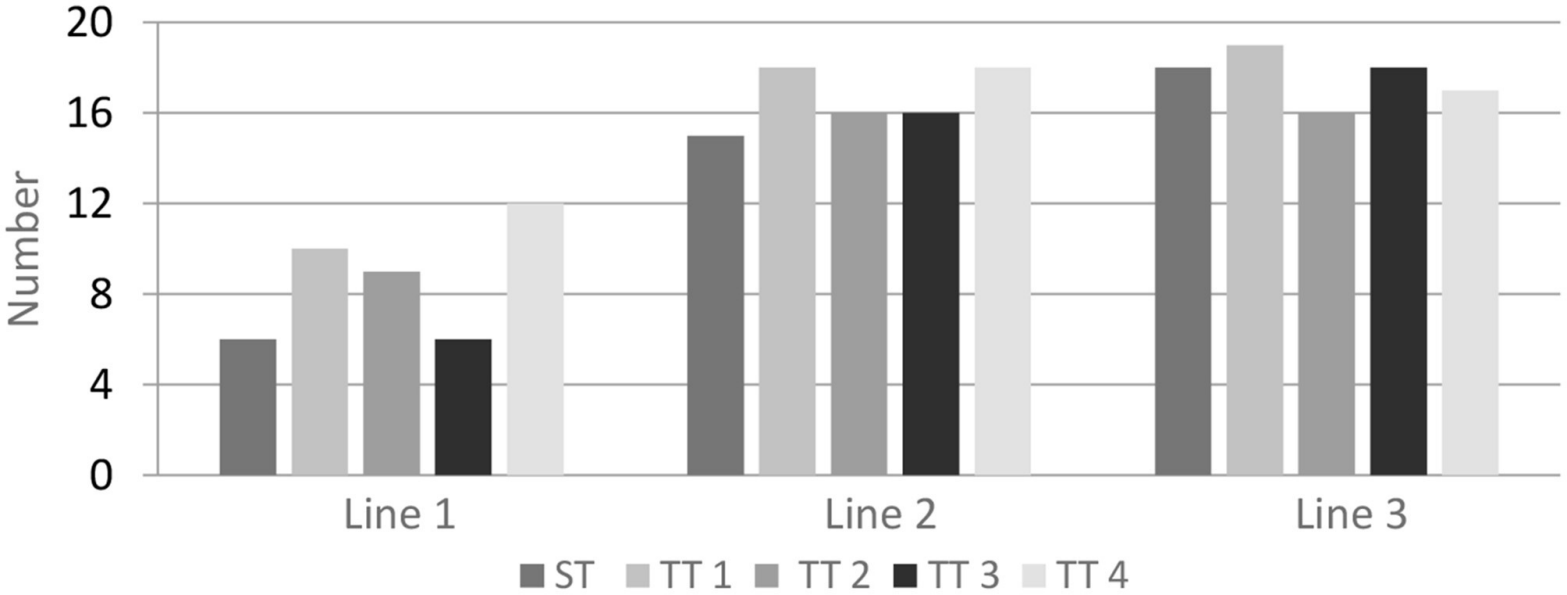
Recognition of imagery superimposition.

Recognition of imagery superimposition in Line 1, Line 2, and Line 3 in the source poem and its four versions of English translations, respectively, can be seen in Figure 2. Columns represent the number of participants who formed connections between mental imageries depicted in each line of each version of the poem.

### Narrative Structure of Imagery

The current study selected the Chinese verb “下”, which means “come” or “go down” in English, as the critical visual word to examine participants' narrative structure of imagery. Both a verbal (the transitivity system; Halliday, 1994) and a non-verbal system (i.e., the visual narrative system; Chen and Li, 2021) are used to categorize participants' responses to the source poem and its four translations. Participants also judged if any forms of imagery were represented in the narrative structures.

As can be seen in Table 1, in the verbal system, all participants judged that the verb “下” is used as the material process (e.g., a process of doing or happening) in all clauses in the poem, as the actor is the “sun”, and in the English translations, verbs including “sinking”, “westering”, “setting”, and “dips down”, as different translations of the Chinese verb “下”, are processes of abstract doing pertinent to the “sun”. However, to represent meaning through imagery, sixteen participants suggested that they could see the dynamic movement of the sunset in the source poem and thought that the day was about to finish (i.e., Action process). Four participants felt more assertive about the representation of the sun instead of the sunset (Reactional process). These variations between individual participants existed in all four versions of the English translations of the source poem. Furthermore, ten participants mentioned they could find the related process in the verbal system of TT1 due to the adding verb “trudging toward”. They argued that the choice of this verb phrase was ineffective in translation, for it reduced the dynamic effect of the sun and could not enhance the logic-semantic relation in the poem.

**Table 1:** Imagery comparison in verbal and non-verbal systems.

**Poem**	**Text (verbal system) transitivity process type**	**Imagery (non-verbal system) narrative structure**
ST: 夕阳西下，断肠人在天涯	Material process (20)	Action process (16)/reactional process (4)
TT1: Trudging toward the ***sinking*** sun, farther and farther away from home.	Material process (20)	Action process (10)/reactional process (10)
TT2: The sun ***westering***, And one with breaking heart at the sky's edge.	Material process (20)	Action process (11)/reactional process (9)
TT3: The sun is ***setting***, Broken man far from home roams and roams	Material process (20)	Action process (15)/reactional process (5)
TT4: The sun ***dips down*** in the west, and the lovesick traveler is still at the end of the world.	Material process (20)	Action process (9)/reactional process (11)

### Salience of Imagery

Participants were asked to determine the salience of the visual words from both the ST and TTs and indicate how they were achieved. Multiple explanations were allowed in their answers.

As can be seen in Table 2, the salience values observed from the visual words representing imagery in the poems varied considerably between participants. In the ST, the ways to achieve the representational imagery of “夕阳” (sunset in English) are the largest. Participants reported that they could imagine the position in the west, the enlarging size, and even the golden color of the sun, enjoying its warmth and beauty, or feeling the passing of the day. But in the TTs, most participants could only feel the setting sun in position, for all the verbs showed a dynamic movement of the sun. Then, the majority of the participants suggested that the most significant visual element in ST was “断肠人” (a heart-broken man), and most of them chose “cultural factor” as its way to achieve its function and considered this element to be the embodiment of traditional Chinese culture. Because “断肠人” is a frequently used literary expression in Chinese poems, often to describe the overwhelming emotional stimulation with the feeling of extreme sadness. Lack of a culture-based understanding of this expression, readers have to spend more cognitive effort when forming imagery of it. Nine participants formed imagery with an unfortunate person from its literal meaning “break one's intestine” (getting people hurt).

**Table 2:** Distribution of imagery salience of subjects.

**Text**	**Salience**	**Ways to achieve**
ST	夕阳 (8)	Cultural factor (8) position (6) size (1) color (2)
	断肠人 (8)	Cultural factor (19)
	天涯 (4)	Cultural factor (14) position (4)
TT1	Trudging toward (5)	Position (2)
	Sinking sun (9)	Cultural factor (8) position (7) size (1)
	Farther and farther (6)	Position (19)
TT2	Sun westering (7)	Position (18)
	Breaking heart (9)	Cultural factor (19)
	The sky's edge (4)	Position (11) cultural factor (5) size (1)
TT3	Sun is setting (5)	Position (13) size (4)
	Broken man (13)	Cultural factor (18)
	Roams and roams (2)	Position (17)
TT4	Sun dips down (6)	Position (18)
	Lovesick traveler (9)	Cultural factor (2) position (3)
	End of the world (5)	Cultural factor (14) position (5)

As compared to ST, TTs correspond to a more significant variation in readers' perceptions of their mental imagery in terms of salience values. People or objects often generate the most considerable imagery elements, but some readers tend to “see” the action details referring to the narrative process. For example, 18 participants perceived the imagery salience of TT2 as “westering” which is a more dynamic expression of a pleasant or sad state of mind than the sunset action itself. Interestingly, in TT 4, nine participants gave salience to “lovesick traveler,” but only two chose “cultural factor,” and three chose “position.” As they could not find any relevance of this expression to the poem's theme, it represented the wrong imagery compared to other visual words in the poem. The same case happened in TT1's “trudging toward” which misled participants more to focus on the action of people instead of the sunset imagery. Regardless of the degree to which different visual words are connected to each other, salience value can create a hierarchy of importance among elements, selecting some as more important, more worthy of attention as compared to others (Kress and Van Leeuwen, 2021). As a principal function of sound in the poem, the rhyme will also give different salience to strengthen the effect of imagery and the sense of beauty. After reading all the poems aloud, fourteen participants reported that they had successive sensations of salience about the stressed syllables and the regular rhyme in ST and TT3, which are more poetic. Because the rhythmic features of poems are not the focus of this paper, these data were not analyzed further.

### Emotive Validity From Imagery

Participants were asked to choose the theme emotion of the poem after reading ST and four translations (multiple choices were available). Most participants could feel the nostalgia and the sadness in the poem, but it was still hard for the five of them, who knew less about Chinese poetry, to construct a complete picture in their minds, such as the sunset scenery in autumn. Nine participants found calmness, positioning themselves for the poem's second line, which describes the peaceful life through three images: bridge, stream, and homes. Participants also expressed their emotional responses to each salient element from Table 2, and the types of emotions all came from the genuine feelings that may arise from representing the meanings of single imagery (see Table 3).

**Table 3:** Emotional response of subjects to the salient imagery.

**Text**	**Salience**	**Emotional response**
ST	夕阳 (8)	Joy (6) interest (5) sorrow (6)
	断肠人 (8)	Sorrow (14) horror (3)
	天涯 (4)	Loneliness (12) sorrow (8)
TT1	Trudging toward (5)	Tired (12) sorrow (6)
	Sinking sun (9)	Calmness (12) sympathy (7)
	Farther and farther (6)	Anxious (5) loneliness (6) sorrow (7)
TT2	Sun westering (7)	Calmness (16) sympathy (4)
	Breaking heart (9)	Sorrow (18)
	The sky's edge (4)	Loneliness (11) sorrow (8)
TT3	Sun is setting (5)	Calmness (16) sympathy (2)
	Broken man (13)	Sorrow (15) confusion (3)
	Roams and roams (2)	Loneliness (5) boredom (8) anxious (6)
TT4	Sun dips down (6)	Calmness (8) sympathy (10)
	Lovesick traveler (9)	Confusion (16) sympathy (2) sorrow (2)
	End of the world (5)	Loneliness (8) hopelessness (9) sorrow (3)

**Figure 3:**
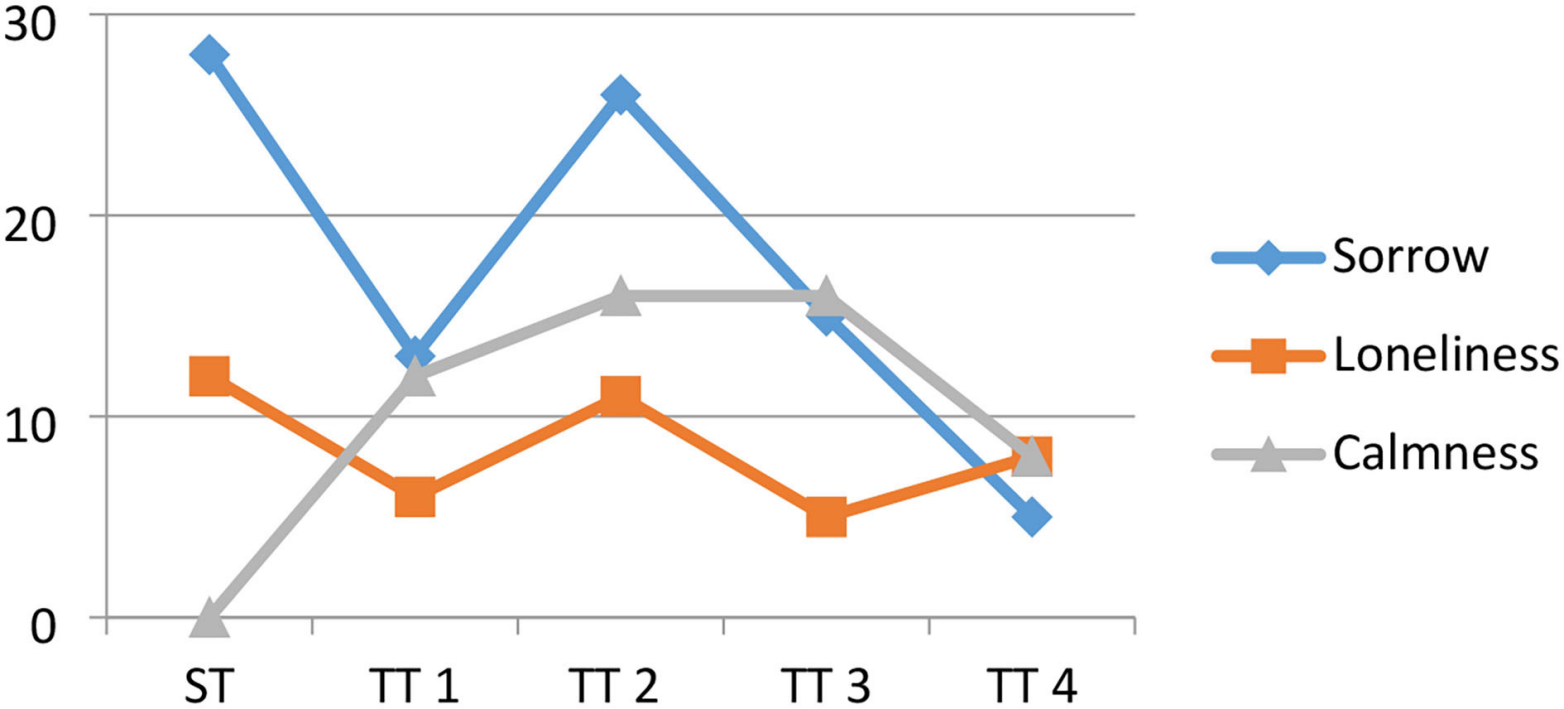
The number of the main emotions in different versions of the poem.

As seen in Table 3, most participants chose “sorrow” for the miserable traveler in ST, TT2, and TT3, which corresponds to the poem's theme. Nevertheless, 2 of them chose “sorrow” for “lovesick traveler”; they suggested that the traveler was not lovesick but homesick, indicating that mistranslated imagery words cannot evoke the same emotional response. Moreover, words with Chinese cultural characteristics, such as “天涯” in ST, are also confused by readers who are not so familiar with Chinese literary works. Thus, the imagery from “at the sky's edge” and “at the end of the world” brings readers different emotional experiences. The verbs as salient elements in ST and TTs failed to deepen readers' emotional experience. As shown in Figure 2, the emotive validity is based on the affective appeal of the salient imagery words, and the imagery words in translated poems do not always convey the same emotive validity as the original poem. The predominant emotional tone of the original is sorrow. By adding up all the numbers of sorrow responses in Figure 3, ST almost realizes the emotive validity according to its background. However, the validity of such a feeling has decreased a lot in TT1, TT3, and TT4. The second emotional factor of a poem is loneliness, which is represented similarly in the four English versions of the poem, and TT2 almost achieves its emotive validity. Finally, calmness, which is not apparent in the original poem, is reflected in participants' reports to varying degrees in the four translated versions, which may violate the validity of the original affection.

## Discussion

The evocation of mental imagery and emotions can assist in understanding and appreciating poetry in different cultural backgrounds. The recognition that target readers access both verbal and non-verbal imagery when reading poems is essential in selecting translation strategies. These strategies can exploit non-verbal aspects (e.g., imagery) of comprehension with target readers whose vocabulary, linguistic, and cultural knowledge of the poem's native language is rather limited. From a psychological point of view, imagery transformation is the process of selecting and matching the imagery to languages. In addition to the multiple influences of the translator's individual variables, such as knowledge, experience, and cognitive style, the conversion is also subject to external factors, such as social, cultural, and thinking traditions. Compared with the aesthetic imagery presented in the original poem, what is restored and reproduced by the translator may be the same, similar, or even wholly different “imagery.”

The findings of the current study showed that the cognitive transformation of imagery contains considerable individual variances. Therefore, the ultimate goal of poetry translation is to achieve imagery-to-imagery equivalence, which refers to the fact that the imagery created has the same semantic and aesthetic implications in the translated versions as in the original language, based on the commonality of thinking and psychological identity. Although different cultures have distinct languages and ways of thinking, there are similarities in understanding the same thing and the translator's style of thinking, processing, and aesthetic experience. As a result, the “meaning” and “imagery” can be connected in the original as in translated languages. At this point, the translator will utilize more image-based reasoning to extract pictures of particular items in the original language, as well as the visual implications that go along with them. Translators will also search the translation system for objective items that fit the meaning and emotions so that they can link the two.

For the imagery in translation works to have the same or similar emotional effect like the one in the original, “image for image” strategy can also be used. This strategy refers to searching for different imagery in the target culture to replace the original one with the translator's imagination. Due to external environmental factors, the materials or information available for people's imagination might be different. That is to say, the equivalent image in the translated context cannot be found to represent the “image” in the original poem to a perfect level. However, since human beings have a high degree of consistency in their understanding of objective things in nature, the content of thinking (the “meaning” in imagery) will stay the same or comparable even if the output of thinking (language) changes in imagery reduction (Zoltan, 2000).

Furthermore, our results suggest that extracting the translated picture with the same visual impact from memory is not always possible. To be “faithful” to the original poem or let the target readers understand the exotic flavor, the translator needs to retain or highlight the imagery of the original work. The semantic or formal equivalence between the “original image” and the “translated image” may be obtained through this process, and the translator must make the meaning of the image explicit by adding notes or supplementary explanations to convey the original imagery's meaning and emotion. Finally, sometimes it is necessary to discard the image to reach the meaning, which means that the translator can ignore images developed in the original poem entirely and only look for the visual word with a similar meaning or effect. This strategy prevents the tautologies of adding long explanations or notes for imagery words in the original poem. This strategy also releases the translator from the burden of looking for expressions in the target language to restore and reproduce the original imagery. The translator may choose to abandon the imagery words in the transformation process based on his aesthetic judgment.

Our findings suggest that the theoretical framework of visual narrative provides a new perspective for analyzing poetry translation. First, we can comprehensively and systematically compare different visual narratives between the original and translated poems. Second, we can explain in more depth how visual imagery regulates readers' emotional engagement and attracts their interests by selecting visual words with almost the same imagery effect and providing theoretical guidance and practical strategies for poetry translation. The concepts of “narrative process,” “salience value,” and “emotive validity” help examine the readers' representational imagery from reading both the source poem and the translated poems. It can be found that there are interpersonal differences in recognizing imagery through visual words and between parallel poems involved in translation because meanings arise in social contexts and personal interaction, which are variables of cultural backgrounds (Kress and Van Leeuwen, 2021).

Imagery in poetry is a vivid and vibrant form of description that produces visual effects and appeals to the readers' senses and imagination (Lewis, 1984). Many translation strategies are studied from culture, linguistics, and translation studies in order to enhance the reproduction of imagery that is presented in target poems. However, some cognitive differences can still be described concretely through the principles of visual grammar. Thus, it is also confirmed that visual grammar can effectively serve as a reference mechanism for a particular reader to construct imagery meaning and evaluate different translations or present different readers' perceptions of the same poem. The poem selected for the current, “天净沙·秋思”, is a classic work of Chinese poetry, and research on its English translations has been conducted extensively. This study attempts to describe the readers' imagery in poetry translation using visual language, peeking into the involvement of verbal and non-verbal systems in translation. Accordingly, it is argued that more intrinsic connections and interactions between verbal and non-verbal systems can be explored in translation studies in conjunction with examining mental imagery.

Imagery in poetry is uncertain in different cultures; it is not as solid and constant as real images, and they vary from one individual to another. The purpose of observing visual words and imagery is not to solve the problem of uniformity but rather to investigate them in-depth as the subjectivity of the cognitive process of translation triggers further studies. Reading poems with high imagery content can be seen as a visualization of words in the reader's mind. Visual grammar offers a new method to explore the interaction between the verbal and the visual, the semiotic and the non-semiotic, and between individual expression and social semiosis.

## Data Availability Statement

The raw data supporting the conclusions of this article will be made available by the authors, without undue reservation.

## Author Contributions

YY and TG contributed to the conception of the study. YY conducted the experiments and drafted the manuscript. TG contributed to the revision of the manuscript. All authors have approved the final version of the manuscript.

## Funding

This research was supported by National Social Science Foundation of China (16BYY008).

## Conflict of Interest

The authors declare that the research was conducted in the absence of any commercial or financial relationships that could be construed as a potential conflict of interest.

## Publisher's Note

All claims expressed in this article are solely those of the authors and do not necessarily represent those of their affiliated organizations, or those of the publisher, the editors and the reviewers. Any product that may be evaluated in this article, or claim that may be made by its manufacturer, is not guaranteed or endorsed by the publisher.

## Footnotes

^1^The HSK (Level V) assesses test takers' abilities in the application of everyday Chinese. It is the counterpart of the Level V of the Chinese Language Proficiency Scales for Speakers of Other Languages and the C1 Level of the Common European Framework of Reference (CEF). Test takers who are able to pass the HSK (Level V) can read Chinese newspapers and magazines, enjoy Chinese films and plays and give a full-length speech in Chinese (see http://www.chinesetest.cn).

## Appendix

Source Text

天净沙·秋思

(元)马致远

枯藤老树昏鸦，小桥流水人家，古道西风瘦马。夕阳西下，断肠人在天涯。

                                           Target Text 1

                                           Autumn

by Ma Chi-yuan

Crows hovering over rugged old trees wreathed with rotten vine—the day is about done. Yonder is a tiny bridge over a sparkling stream, and on the far bank, a pretty little village. But the traveller has to go on down this ancient road, the west wind moaning, his bony horse groaning, trudging towards the sinking sun, farther and farther away from home.

                                                 –tr. by Weng Xianliang

Target Text 2

                                           Tune to"Sand and Sky"

                                           —Autumn Thoughts

                                           by Ma Zhiyuan

Dry vine,old tree,crows at dusk,
  Low bridge,stream running,cottages,
  Ancient road,west wind,lean nag,
  The sun westering
  And one with breaking heart at the sky's edge.

                                                 -tr.by Wayne Schlepp

Target Text 3

                                           Autumn Thoughts

                                           by Ma Zhiyuan

Withered vines, olden tree, evening crows;
  Tiny bridge, flowing brook, hamlet homes;
  Ancient road, wind from west, bony horse;
  The sun is setting,
  Broken man, far from home, roams and roams.

                                                 -tr.by Zhao Zhentao

Target Text 4

                                           Tune:Tian Jin Sha

                                           by Ma Zhiyuan

Withered vines hanging on old branches,
  Returning crows croaking at dusk.
  A few houses hidden past a narrow bridge,
  And below the bridge a quiet creek running.
  Down a worn path,in the west wind,
  A lean horse comes plodding.
  The sun dips down in the west,
  And the lovesick traveller is still at the end of the world.

                                                 -tr.by Ding Zuxin and Burton Raffel.
